# Customized strategies for high-yield purification of retinal pigment epithelial cells differentiated from different stem cell sources

**DOI:** 10.1038/s41598-022-19777-2

**Published:** 2022-09-16

**Authors:** Kakkad Regha, Mayuri Bhargava, Abdurrahmaan Al-Mubaarak, Chou Chai, Bhav Harshad Parikh, Zengping Liu, Claudine See Wei Wong, Walter Hunziker, Kah Leong Lim, Xinyi Su

**Affiliations:** 1 Institute of Molecular and Cell Biology (IMCB), Agency for Science, Technology and Research (A*STAR), Singapore, 138673 Singapore; 2 Department of Ophthalmology, Yong Loo Lin School of Medicine, National University of Singapore (NUS), Singapore, 117597 Singapore; 3 Department of Ophthalmology, National University Hospital (NUH), Singapore, 119074 Singapore; 4 Lee Kong Chian School of Medicine, Nanyang Technological University (NTU), Singapore, 308232 Singapore; 5 Singapore Eye Research Institute (SERI), Singapore, 169856 Singapore; 6 Department of Physiology, Yong Loo Lin School of Medicine, National University of Singapore (NUS), Singapore, 117593 Singapore

**Keywords:** Stem-cell differentiation, Induced pluripotent stem cells, Embryonic stem cells

## Abstract

Retinal pigment epithelial (RPE) cell dysfunction and death are characteristics of age-related macular degeneration. A promising therapeutic option is RPE cell transplantation. Development of clinical grade stem-cell derived RPE requires efficient in vitro differentiation and purification methods. Enzymatic purification of RPE relies on the relative adherence of RPE and non-RPE cells to the culture plate. However, morphology and adherence of non-RPE cells differ for different stem cell sources. In cases whereby the non-RPE adhered as strongly as RPE cells to the culture plate, enzymatic method of purification is unsuitable. Thus, we hypothesized the need to customize purification strategies for RPE derived from different stem cell sources. We systematically compared five different RPE purification methods, including manual, enzymatic, flow cytometry-based sorting or combinations thereof for parameters including cell throughput, yield, purity and functionality. Flow cytometry-based approach was suitable for RPE isolation from heterogeneous cultures with highly adherent non-RPE cells, albeit with lower yield. Although all five purification methods generated pure and functional RPE, there were significant differences in yield and processing times. Based on the high purity of the resulting RPE and relatively short processing time, we conclude that a combination of enzymatic and manual purification is ideal for clinical applications.

## Introduction

Retinal pigment epithelium (RPE), a monolayer of hexagonal cells, forms the outer blood-retinal barrier and plays an important role in retinal homeostasis^1^. RPE dysfunction is a feature of retinal degenerative diseases such as age-related macular degeneration (AMD), or Stargardt’s disease for which there are currently no treatments^2^. Replacing non-functioning RPE with healthy cells could be a promising therapeutic approach for these diseases^3,4^.

Stem cell derived RPE cells are a potential source for retinal cell therapy. RPE cells derived from pluripotent stem cell sources such as embryonic stem cells (ESC) and induced pluripotent stem cells (iPSC) have been used in clinical trials for AMD^2,4–10^. Regardless of cell source, current protocols for generation of RPE result in a heterogenous population also containing non-RPE cells^11–13^. Removal of non-RPE cells is required for clinical trials to ensure reproducibility and safety^6^.

Efficiency of differentiating RPE from stem cells is known to be variable^14^. There are several approaches used for the removal of non-RPE cells from a heterogenous RPE population. These include manual picking or scraping, enzymatic separation and flow cytometry^8,10,14^. For manual purification, pigmented polygonal cells (assumed to be RPE) are selectively picked or non-polygonal non-RPE cells are visually identified and scraped off, whilst retaining RPE cells with polygonal morphology^12,13^. Enzymatic isolation of RPE from mixed cultures relies on the stronger adherence of RPE cells to culture plate compared to non-RPE cells. Non-RPE cells can be easily detached by short exposure to dissociating agents such as Accutase or TrypLE^15–17^. Flow cytometry-based methods take advantage of higher side scattering (SSC) of light due to the presence of pigmentation within the RPE cells^18^. While manual purification does not require specialized reagents or expensive equipment, it is time-consuming and low-throughput as it involves manually picking of RPE cells or scraping off the non-RPE cells under a microscope^12^. Enzymatic purification is easy to perform. Flow-cytometry based methods are more costly due to the need for expensive equipment and may result in lower yield due to higher loss of cells during flow cytometry process^19^.

Although enzymatic purification is easy to perform, it is only useful for cultures in which the non-RPE cells have low adherence to the underlying culture plate relative to the RPE cells. We found that the non-RPE cells in differentiation cultures derived from some stem cells vary in morphology and adherence strength. In some differentiation cultures they formed dome-shaped clusters with low adhesion to the cell culture plate whereas in others the non-RPE adhered strongly just like the RPE cells. We hypothesized that RPE purification approaches will have to be optimized and adapted for various RPE cell products derived from different stem cell sources. We compared five purification methods, including manual, enzymatic, flow cytometry-based sorting or combinations thereof using skin-iPSC- and ESC-derived RPE cultures. We found that all purification methods give highly functional RPE cells, but their yield and processing times vary significantly. Our findings can help researchers to make informed decisions about RPE purification strategies and customize them based on the relative adherence strength of non-RPE and RPE to the culture plate.

## Results

### RPE cultures obtained from different stem cell sources demonstrate heterogeneity in morphology of the non-RPE cells

**Figure 1:**
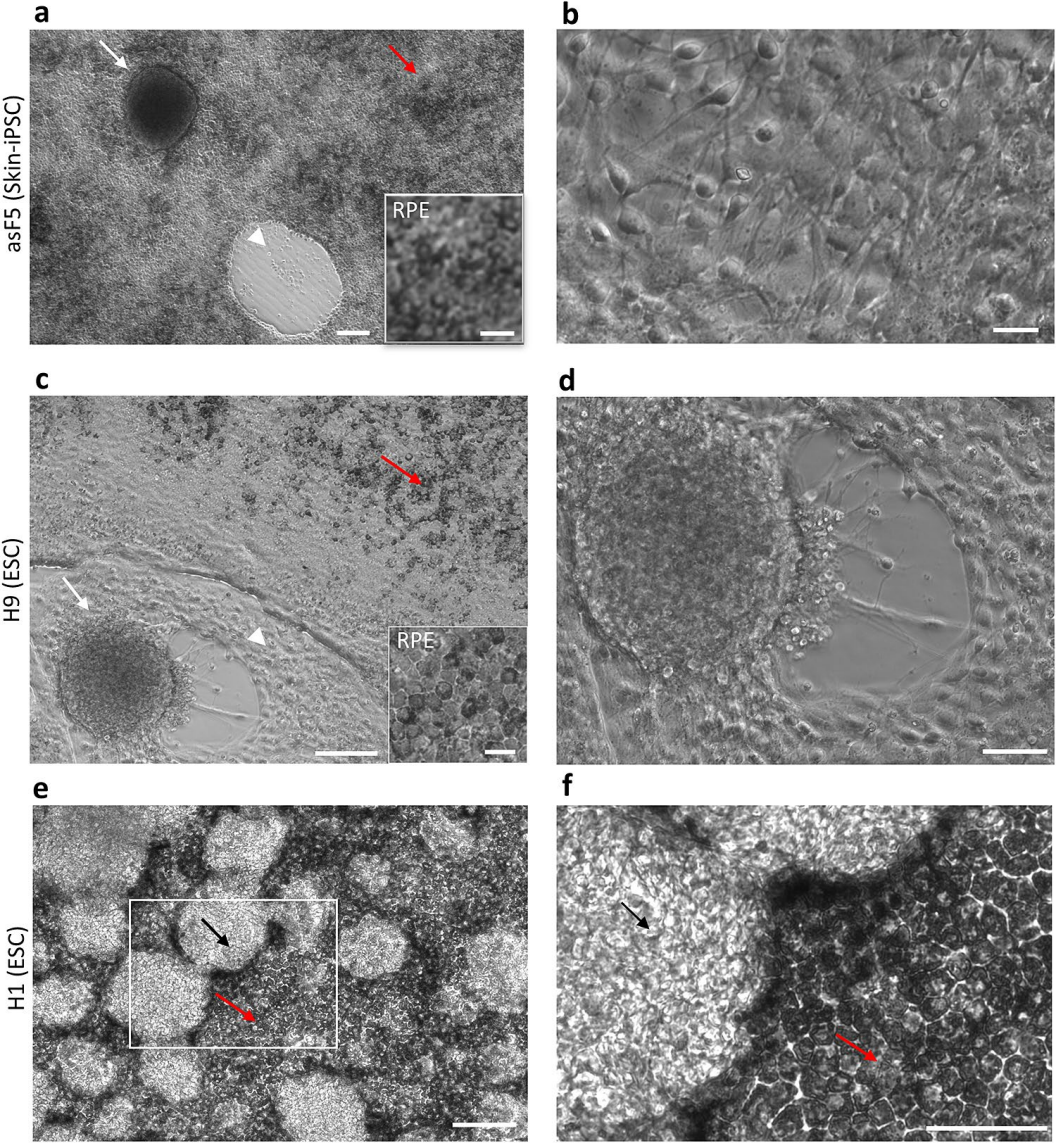
Morphological differences in non-RPE cells from skin-iPSC and ESC sources. (**a**) Dome-shaped clusters (white arrow) of non-RPE cells and empty area formed by detachment of a cluster (white arrowhead) in asF5 (skin-iPSC) differentiation cultures close to a polygonal RPE cell sheet (red arrow, inset). Scale bar, 100 μm. Inset scale bar, 20 μm. (**b**) Magnified view of the non-polygonal non-RPE cells with protrusions. Scale bar, 20 μm. (**c**) Cluster of non-RPE cells (white arrow) surrounded by non-polygonal non-RPE cells (white arrowhead) and RPE cells (red arrow, inset) in H9 (ESC) differentiation cultures. Scale bar, 100 μm. Inset scale bar, 20 μm. (**d**) Magnified view of the non-RPE cells with protrusions. Scale bar, 50 μm. Non-RPE cells (black arrow) in H1 (ESC) differentiation cultures intermingled with pigmented RPE cells (red arrow). Scale bar, 100 μm. Non-RPE clusters (black arrow) in H1 (ESC) differentiation cultures intermingled with RPE cells (red arrow) and non-RPE cells, scalebar 100 μm. (**f**) Magnified view of the area shown inside the white box in (**e**). Scale bar, 50 μm.

RPE was generated from skin-iPSCs (asF5 and HDFa) and ESCs (H9 and H1) sources using a 16-day directed differentiation protocol^12,20^. After 16 days of differentiation, the cells were allowed to further mature and develop pigmentation in RPE maintenance medium for additional 15–20 days. At this stage, differentiated RPE cells in the culture can be identified by their distinctive polygonal shape and pigmentation. Cells not showing these characteristics are identified as non-RPE cells^12,13^. Based on these criteria, we systematically characterized the heterogeneity in all four stem cell derived RPE differentiation cultures (asF5, HDFa, H1 and H9) using light microscopy. In asF5-, HDFa- and H9-derived RPE differentiation cultures, two main types of non-RPE cells were observed, (i) dome-shaped clusters that are formed from multi-layered cells (Figs. 1a,c,d and S1a,b), and (ii) sheets of non-polygonal cells with protrusions (Figs. 1b–d and S1d,e). In phase contrast images, the dome-shaped clusters appeared dark due to opaqueness resulting from the light scattering and dispersion because of the multi-layering phenomenon rather than the presence of pigment granules, a characteristic of RPE cells^21^. In contrast, RPE cells differentiated from H1 cultures did not contain dome shaped non-RPE cell clusters. Instead, they were primarily composed of clusters of non-polygonal cells dispersed within the RPE cell sheet (Fig. 1e,f). In addition, the non-RPE cell clusters in H1 were relatively brighter in phase contrast images.

### Non-RPE cells show heterogeneity in adherence to the underlying cell culture surface

**Figure 2:**
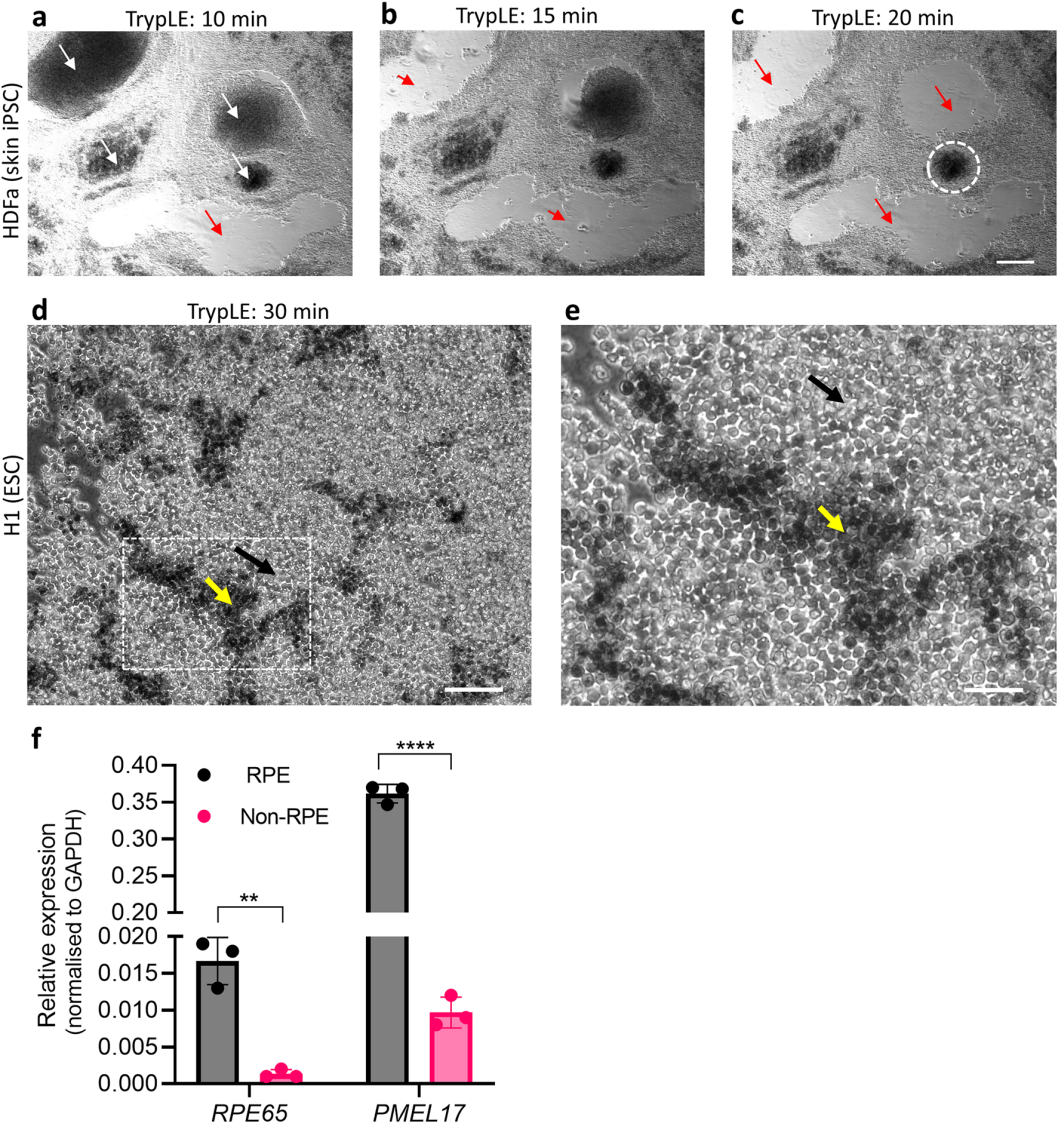
Heterogeneity in adherence of non-RPE cells from different stem cell sources. (**a**) Phase contrast images of HDFa (skin-iPSC) differentiation cultures showing dome-shaped non-RPE clusters of various sizes (white arrows) of darker contrast due to multi-layering rather than pigmentation. (**b** and **c**) Progressive detachment of the non-RPE clusters at 10, 15 and 20 min of TrypLE treatment, leaving gaps (red arrows). Scale bar, 200 μm. Majority of the non-RPE clusters detach within 30 min of treatment except for a small TrypLE-resistant cluster (white dotted circle). (**d**) Non-RPE cells (black arrow) in H1 (ESC) differentiation cultures after 30 min of TrypLE treatment intermingled with pigmented RPE cells (yellow arrow). Scale bar, 100 μm. (**e**) Magnified view of an area inside the box in field shown in (**d**). Scale bar, 50 μm. (**f**) RT-qPCR analysis of weakly and adherent non-RPE and strongly adherent RPE fractions obtained after differential TrypLE treatment of HDFa (skin-IPSC) differentiation cultures for signature genes of RPE. Data represents mean ± s.d. of three replicates for RT-qPCR. Statistical analysis was performed using an unpaired two-tailed students *t*-test (*) = p < 0.05; (**) = *p* < 0.005; (****) = *p* < 0.0001.

Non-RPE cells have a relatively weaker adherence to cell culture plates compared to RPE cells, and sometimes detach during pipetting leaving empty areas on the culture plates (Fig. 1a, white arrowhead). This reduced adherence strength has been harnessed in RPE purification techniques for the preferential removal of non-RPE cells by short treatment with enzymatic dissociation agents, such as TrypLE and Accutase^16,17^. In this study, we examined the adherence strength of non-RPE cells to the growth surface of culture plates generated from all four stem cell lines mentioned previously, using TrypLE dissociation and monitored their detachment using phase contrast microscopy. HDFa was used because the RPE differentiation culture contained an almost equal percentage of both RPE and non-RPE cells, 50.1 and 49.9%, respectively. TrypLE treatment of HDFa cultures resulted in the detachment of both dome-shaped clusters and non-polygonal sheets. By 10–20 min, most of the non-RPE cells have detached, leaving gaps between the strongly adherent RPE cell sheet (Figs. 2a–c and S1c), with minimal small clusters of non-polygonal TrypLE-resistant cells remaining (Fig. 2c, white dotted circle). This was similarly observed in asF5 and H9 (ESC) differentiation cultures (data not shown). By contrast, in H1 (ESC) differentiation cultures, most of the non-RPE cells failed to detach even after prolonged TrypLE treatment for 30 min (Fig. 2d,e). These demonstrate that adherence of non-RPE cells to growth surfaces varies significantly between HDFa and H1 cell types.

To verify that the weakly adherent cells removed after TrypLE treatment in the HDFa differentiation cultures were indeed non-RPE cells, we analyzed the gene expression levels of RPE signature genes (*RPE65* and *PMEL17*) from both the strongly and weakly adherent cell fractions of HDFa differentiation cultures. *RPE65* and *PMEL17* were highly expressed in the strongly adherent fraction, but not in the weakly adherent fraction, with a 12- and 35-fold increase, respectively (Fig. 2f). This further suggests that the weakly adherent cells in HDFa were indeed non-RPE cells, and that TrypLE dissociation was effective in enriching RPE cells.

### Comparison of RPE differentiation culture purification methods for RPE cell yield and purity

**Figure 3:**
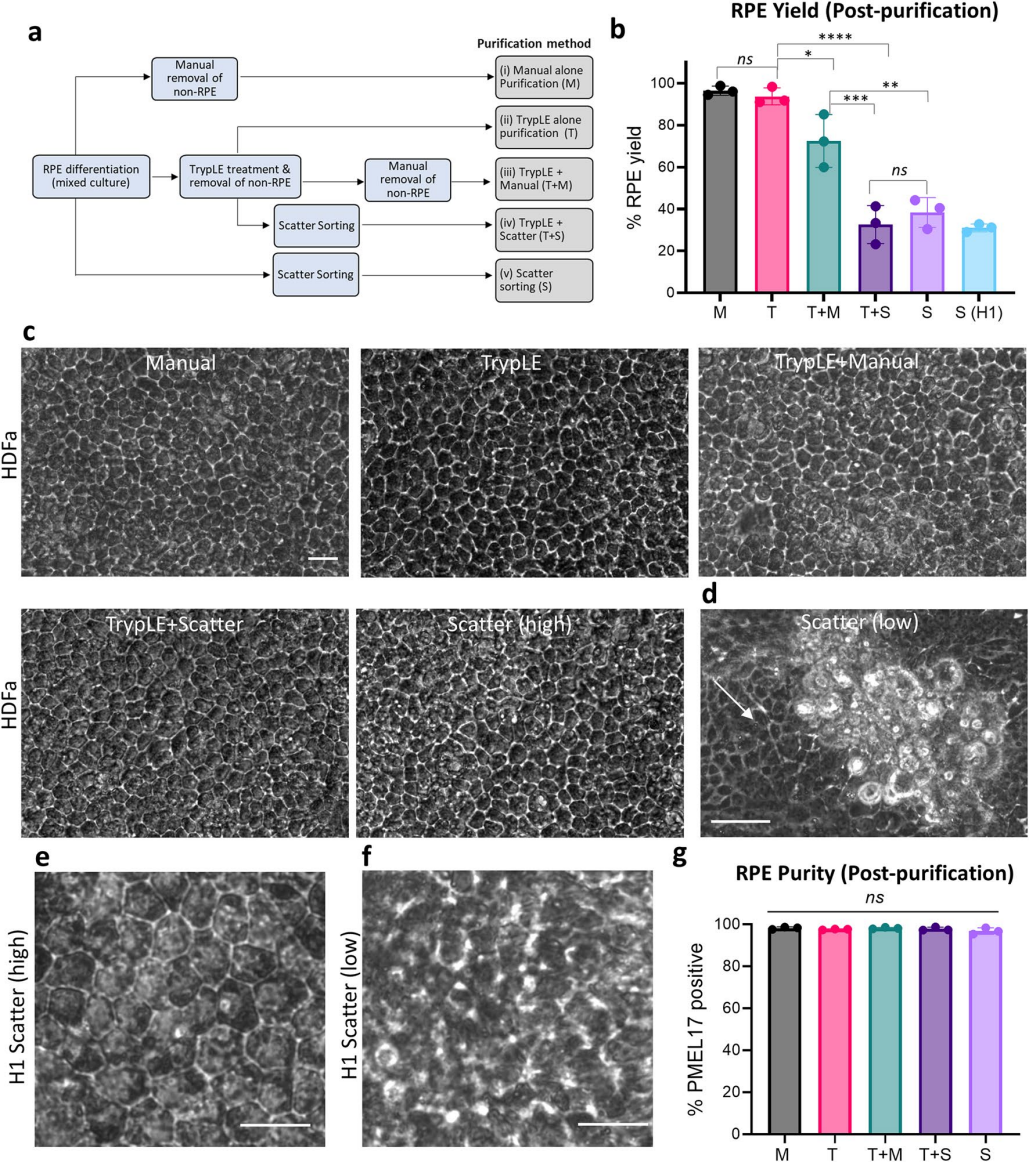
Comparison different RPE purification methods for yield and purity. (**a**) Overview of steps involved in the five different RPE purification methods. (**b**) HDFa- RPE yield of the different purification methods. S (H1): RPE cells from H1 differentiation cultures purified by scatter sorting. (**c**) Purified HDFa-RPE from all five methods were cultured for 6 weeks and showed typical polygonal morphology. Scale bar, 20 μm. (**d**) Cells obtained from scatter low fraction post-sorting showed clusters of non-RPE cells, and polygonal RPE cells (white arrow). Scale bar, 50 μm. (**e** and **f**) 6 weeks old cultured H1-RPE cells obtained from (**e**) scatter high and (**f**) low fractions of H1 (ESC) differentiation cultures. Scale bar, 20 μm. (**g**) Purity of cells obtained by different methods assessed by PMEL17 flow cytometry after 6 weeks in culture. Statistical analysis for (**b**) and (**g**) done using one-way ANOVA, followed by Tukey’s honest significance difference (HSD) post hoc test. *ns*, not significant, (*) = *p* < 0.05; (**) = *p* < 0.005, (***) = *p* < 0.0005, (****) = *p* < 0.0001.

To determine an efficient purification strategy tailored towards the elimination of a specific form of non-RPE cells, five different methods were compared, (i) manual alone purification (M), (ii) enzymatic purification using TrypLE (T), (iii) combination of TrypLE and Manual scraping (T + M), (iv) combination of TrypLE and scatter sorting (T + S), (v) scatter sorting alone (S) (Fig. 3a). To estimate the purification efficiency of the different methods, we used HDFa differentiation cultures as they contained a mixture of RPE and non-RPE cells containing 50.1% RPE cells.

Methods ‘M’ and ‘T’ gave high percentage yields, with an average of 96.5 and 93.7%, respectively (Fig. 3b and Table 1). The average RPE yield from method ‘T + M’ was 72.5%. The significantly lower yield in ‘T + M’ compared to ‘M’ and ‘T’ alone purifications was likely due to loss of some weakly attached RPE cells during manual purification due to a weaker attachment to the culture plate because of the prior TrypLE treatment. Lower average RPE yields were observed with methods ‘S’ (38.4%) and ‘T + S’ (32.6%), likely due to cell loss during scatter sorting^21^. These results indicate that manual and TrypLE alone were the most effective methods for enrichment of RPE cells.

**Table 1:** Comparison of RPE purification methods.

Feature	Manual picking (M)	TrypLE alone (T)	TrypLE + manual (T + M)	TrypLE + scatter (T + S)	Scatter (S)
Average RPE yield (%)	95–99	91–98	60–85	23–41	30–44
Post-purification RPE purity (PMEL17%)	98.2	97.6	98.2	97.8	96.8
Processing time in minutes for 2 wells of a 6-well plate with 50.1% RPE	90–100	30–40	45–60	60–70	100–120
Cell throughput	Low	High	Moderate	Moderate	Low

As both the RPE and non-RPE cells in H1 differentiation cultures had similar adherence to the culture plate, purification of RPE cells by partial TrypLE treatment was not feasible. Manual purification was also not ideal as non-RPE clusters were uniformly intermingled within the RPE cells (Fig. 1e,f) making their manual removal technically challenging. Hence, we tested the suitability of SSC-based flow cytometry cell sorting and obtained an average RPE yield of 31.0% (Fig. 3b).

To determine the purity of RPE cells obtained using the various purification methods, we performed phase contrast microscopy and PMEL17 flow cytometry with purified HDFa-RPE grown for 6 weeks to allow further maturation. We observed that all purification methods obtained RPE cells of polygonal morphology indicating that all strategies used effectively removed non-RPE cells (Fig. 3c). A mixture of polygonal (RPE) and non-polygonal (non-RPE) cells were observed in the scatter low fractions after SSC sorting of HDFa cultures (Fig. 3d), suggesting weakly pigmented RPE cells were lost in the scatter low fraction, which correlated with the lower RPE yield observed previously. In contrast, scatter high populations of H1 differentiated cells showed polygonal cells, and scatter low populations of non-polygonal cells, confirming they were non-RPE (Fig. 3e,f), suggesting that scatter sorting is an ideal purification strategy for differentiation cultures, such as H1, whereby both RPE and non-RPE cells adhere with similar strength to the growth surface. The purity of HDFa RPE obtained from different methods was further verified by culturing the cells for 6 weeks and performing PMEL17 flow cytometry. All methods gave > 96.8% PMEL17 positive fraction, confirming presence of highly pure and stable RPE cell populations (Fig. 3g and Table 1). To further ensure the purity of the RPE, we looked for the presence of other markers specific to non-RPE cell types such as pluripotent (*LIN28* and *OCT4*), mesenchymal cells (Fibronectin and *SNAIL2*) and endodermal cells (*SOX17*) by qPCR and found them to be absent in the purified cells (Fig. S2)^20,22^. This further confirms the purity of HDFa-RPE cells, consistent with the PMEL17 flow cytometry results.

The total time required for the completion of the various RPE purification methods varies. Manual purification of RPE cells from two wells of 6-well culture plate containing 50.1% RPE and 49.9% non-RPE needed approximately 90–100 min (Table 1). Processing time for flow cytometry-based method ‘S’ required 100–120 min for 16 million cells from two wells of 6-well plate, because sorting alone required 40–50 min for a cell suspension with a 10 million cells/mL density. The high processing time needed for methods ‘M’ and ‘S’ significantly reduces their suitability for high throughput cell processing. In contrast, TrypLE purification was fast (30–40 min) and amenable for high throughput RPE purification as many wells could be processed simultaneously. Methods ‘T + M’ and ‘T + S’ had moderate processing time, which may allow high throughput cell processing (Table 1).

### Functional characteristics of purified RPE cells

**Figure 4:**
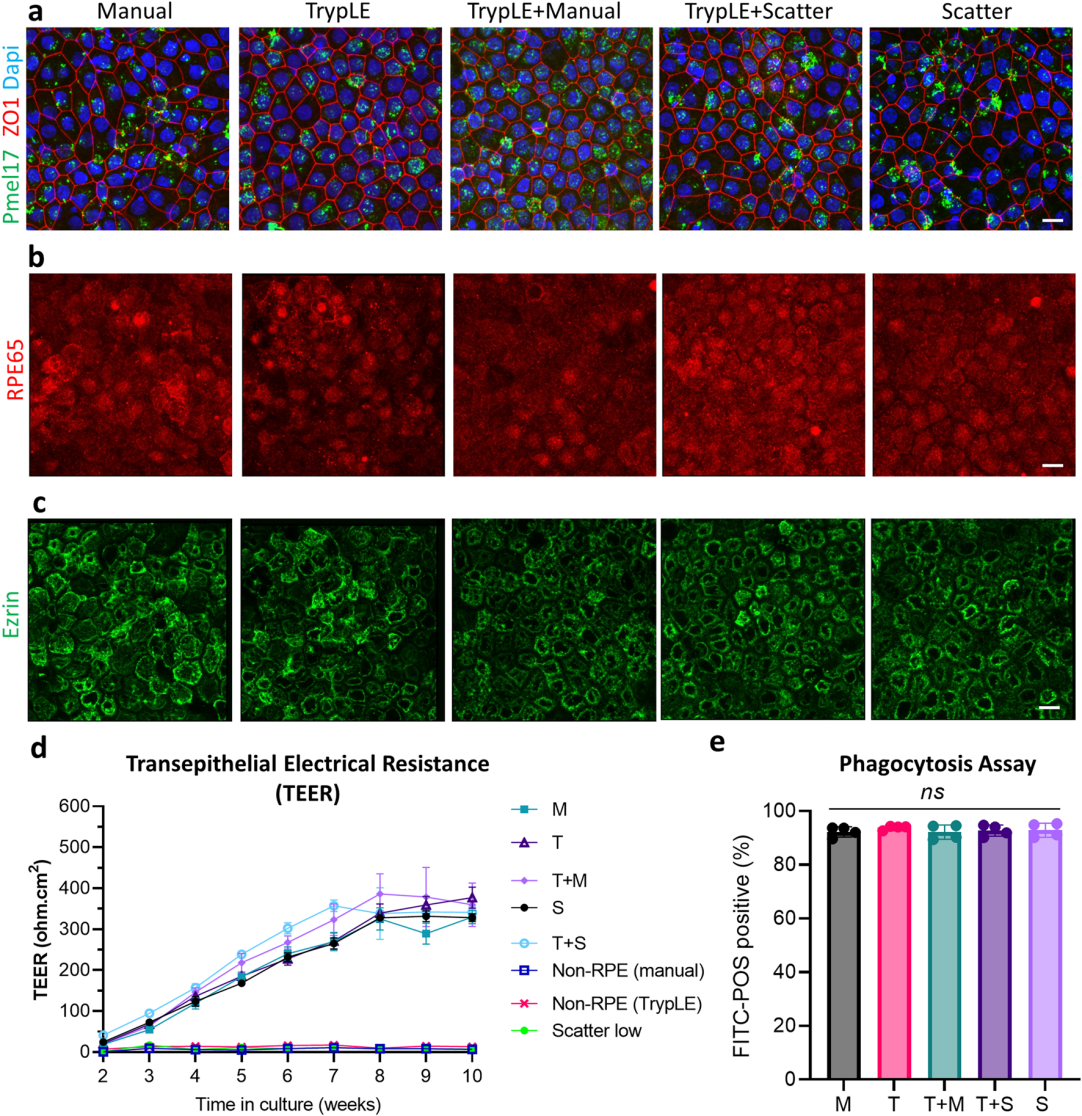
Functional characterization of purified HDFa RPE cells obtained from all methods. (**a**–**c**) Immunostaining for RPE-specific functional proteins, (**a**) zonula occludens-1 (ZO-1) and PMEL17, (**b**) RPE65, and (**c**) Ezrin. Scale bar for **(a**–**c),** 10 μm (**d**) Barrier formation of purified RPE cells and non-RPE was monitored using TEER for 10 weeks and showed progressive increase only for purified RPE cells. (**e**) Functional phagocytosis studied using internalization of FITC-labelled POS and showing > 92.1% uptake for RPEs obtained from all five methods. Statistical analysis was calculated using one-way ANOVA, followed by Tukey’s honest significance difference (HSD) post hoc test. *ns*, not significant.

HDFa-RPE cells purified using various methods expressed PMEL17, a melanosome protein enriched in pigmented cells, and tight junction protein zonula occuldens-1 (ZO-1) (Fig. 4a)^23^. Cytoplasmic expression of retinoid isomerohydrolase (RPE65) and Ezrin, a microvillus enriched protein, were also observed (Fig. 4b,c)^24^. The transepithelial electrical resistance (TEER) was measured as a surrogate for the barrier function of the purified RPE^25^. By week 10, the TEER for RPE derived from the different purification techniques were similar and exceeded 300 Ω cm^2^, consistent with previously reported values for stem cell derived RPE (Fig. 4d)^26,27^. As expected, the TEER was negligible in weakly adherent cells after TrypLE treatment and in SSC low fractions, indicating that they did not contain many RPE (or other epithelial) cells. The phagocytic capacity of purified RPE cells was determined by feeding the cells with FITC-labeled POS and measuring uptake via fluorescence intensity in flow cytometry^28^. All purification methods resulted in RPE cells with > 92.1% FITC-POS uptake, indicating active phagocytosis by these cells (Fig. 4e). Overall, these purified RPE, regardless of the purification method, demonstrated key RPE functions.

## Discussion

The primary goal of this study was to enable researchers to make informed decisions about RPE purification methods for different stem cell sources (iPSC and ESC) by quantifying parameters including cell throughput, yield, purity and functionality. We demonstrated that non-RPE cells from different stem cell derived cultures adhere with varying strengths to the growth surface, enabling a tailored purification strategy. By comparing five different methods of purification for differentiation cultures with heterogeneous adherence of RPE and non-RPE to the culture plate, such as HDFa, we found that Manual (M) or TrypLE (T) alone purifications are effective in obtaining > 91.1% RPE cell yields. For cultures with similar adherence of RPE and non-RPE, such as H1, scatter sorting is ideal. However, it results in a considerable loss of RPE cells during sorting. All five purification strategies investigated in this study gave highly pure populations (> 96.8%) of functional RPE cells that displayed key characteristics of their native counterparts in the eye, including polygonal morphology, expression of RPE-specific proteins, barrier formation and active phagocytosis of photoreceptor outer segments.

Processing time and cell throughput are important considerations in choosing an RPE purification strategy. The processing time for manual purification is high because it involves identification and removal of individual non-RPE cells under the microscope making it a low throughput method. Manual purification of two wells of a 6-well culture plate with a heterogenous mixture of 50.1% RPE and 49.9% non-RPE cells needed 90–100 min. Moreover, the prolonged time that cells have to remain at room temperature during manual purification may also cause stress and cell death. Another disadvantage of the manual method is that the identification and removal of non-RPE cells by morphological appearance under the microscope is subjective, resulting in variable outcomes. In addition, it will be affected by the experience of the researcher and selection criteria that could be ambiguous. In conclusion, manual purification results in high RPE yields and is relatively low-cost since it does not require expensive machinery. However, it is time-consuming, low-throughput and highly variable, due to its reliance on operator’s experience and competency.

TrypLE purification, on the other hand, is a high throughput and fast method, as many wells can be treated with TrypLE simultaneously. This makes it an ideal method for large-scale RPE cell production. In this study, the time required for TrypLE purification of a mixed RPE differentiation culture containing 50.1% RPE from all wells of a 6-well plate was 30–40 min. However, the disadvantages of TrypLE purification are that it does not remove TrypLE-resistant non-RPE cells and is therefore not ideal for clinical applications. In contrast, methods ‘T + M’ and ‘T + S’ can remove TrypLE-resistant non-RPE cells. For clinical applications, ‘T + M’ would be preferred despite the relative lower yield of 60.0–85.2% because of the ability to obtain high purity (98.2%), with a reasonable processing time of 45–60 min. Of note, flow cytometry-based method ‘S’ is time consuming with 10 million cells requiring a minimum of 100–120 min in total including cell sorting. The low throughput and low yield of method ‘S’ make it an unsuitable method for large-scale RPE cell purification. Moreover, cell sorting has been known to be associated with generation of oxidative stress, mitochondrial damage, and apoptosis of the sorted cells^29^. Different extracellular matrix coatings or RPE differentiation protocols may theoretically influence the type and adherence strength of non-RPE cells in the final RPE differentiation culture. Previous studies have consistently demonstrated the presence of weakly adherent non-RPE cells that can be detached more easily with Accutase or TrypLE treatments, regardless of alternative coating materials (such as laminin) or differentiation protocols^16,17^. This suggests that the purification methods described in this study would be relevant and applicable to other RPE differentiation methods and cell lines.

In this manuscript, we described customized RPE purification strategies to ensure maximal cell yield and purity for different stem cell resources and proposed that a combination of enzymatic and manual method of purification would be most suitable for clinical applications. Another key finding of our study is that RPE purification methods would have to be customized for RPE derived from different stem cell cultures. The relative strength of adherence of non-RPE cells to the cell culture plate, should be an additional consideration as one selects for a suitable purification method.

## Methods

### Stem cell culture

The iPSCs were generated from human dermal fibroblasts HDFa (Cascade Biologics) and asF5 (CellResearch Corporation Pte Ltd). Somatic reprogramming of skin fibroblasts was performed using a modification of the previously described reprogramming method^30^. The episomal vectors pCXLE-hOCT3/4-shp53-F, pCXLE-hSK, and pCXLE-hUL were purchased from Addgene (27077, 27078, 27080, respectively). Actively growing fibroblasts were co-transfected with the three vectors using the Neon Transfection System. The iPSC lines were maintained feeder-free in mTeSR1 (StemCell Technologies 85850). Human ESCs (H1 and H9) were cultured as described previously^20^.

### RPE differentiation and culture

The iPSC (HDFa and asF5) and ESC (H1 and H9) were differentiated into RPE using published protocol with minor modifications^12,20^. For differentiation, the stem cells were grown to 90% confluency in mTeSR1 on six-well culture plates coated with Matrigel (Corning 354230). At the end of differentiation (Day 16), the medium was switched to RPE maintenance medium^31,32^ (termed RPE medium). Differentiated iPSC- and ESC-derived RPE cultures were maintained at 37 °C under 5% CO_2_ and medium was changed every 2–3 days. Purified RPE cells were grown in cells culture plates (Corning) coated with Synthemax-II (Corning 3535). For applications such as TEER and phagocytosis assay cells were grown on Transwell cell culture inserts (0.4 μm pore size, 3470 Corning) coated with Synthemax-II. RPE cells (4–6 weeks old) were passaged using 1X TrypLE Express (Gibco, 12604013) for 30 min.

### Reverse transcription quantitative real-time polymerase chain reaction (RT-qPCR)

Total RNA from RPE cultures were isolated using RNeasy Mini Kit (Qiagen)The cDNA was prepared from1 μg RNA using iScript cDNA Synthesis Kit (Bio-Radd, 1708841). The qPCR was done with gene-specific primers (Table S1) using KAPA SYBR FAST qPCR Master Mixt (Sigma-Aldrich, KK4618) on QuantStudio 5 Real-Time PCR system. Data was analyzed by comparative CT method and normalized to *GAPDH*.

### RPE purification methods

30–35 days old RPE differentiation cultures were used for all following methods of purifications, by when the RPE cells were easily identifiable as pigmented polygonal cells under the microscope.

#### Manual alone purification (M)

The differentiation cultures were observed under the dissection microscope to identify the non-RPE cells based on their absence of pigmentation, dome-shaped appearance, or non-polygonal shape. They were removed manually by scraping them off with a 10 μL tip attached to micropipette by observing the culture plate under a dissection microscope. The plate was washed three times with 1X phosphate-buffered saline (PBS) to remove the non-RPE cells. The remaining cells consisting of RPE, were dissociated using TrypLE and incubating for 30 min at 37 °C.

#### Enzymatic alone purification using TrypLE (T)

The differences in adherence strength of RPE and non-RPE cells to the culture plate was utilized in the enzymatic purification. Due to the weaker attachment to the growth surface, non-RPE cells were removed by shorter treatment time to dissociation agent (TrypLE) for 10–20 min, compared to RPE cells (adherent to the culture plate even after 30 min, but dissociated to single cells when pipetted). The culture medium was aspirated, and cells were washed once with PBS and incubated with TrypLE for 10 min at 37 °C (1st TrypLE treatment), regularly observing the cultures under the microscope for floating non-RPE clusters. The culture plate was gently swirled or tapped to aid the detachment of weakly adherent non-RPE clusters which were aspirated and discarded or collected as the “non-RPE” cell fraction for gene expression studies. The TrypLE treatment was repeated 2 more times (2nd and 3rd TrypLE treatment), 5 min each and the non-RPE cells were removed by aspiration. The plate was gently washed twice with PBS to remove non-RPE cells. The RPE cells, still attached weakly to the culture plate, were dissociated by incubating with fresh TrypLE for additional 10 min (4th TrypLE treatment) followed by vigorous pipetting.

#### TrypLE + manual purification (T + M)

Majority of the non-RPE clusters were removed by TrypLE treatment as described above. TrypLE resistant non-RPE clusters were seen at low frequency and were removed by manual purification as described in Manual alone purification.

#### TrypLE + scatter sorting (T + S)

The non-RPE clusters were first removed by TrypLE treatment as described before. The remaining cells were dissociated to single cells by further TrypLE treatment for 10–15 min and purified by flow cytometry-based scatter sorting (SSC) to remove the TrypLE resistant non-RPE cells as described previously^18^. The non-RPE cells removed by initial TrypLE treatment were used to set the scatter low gating during flow cytometry.

#### Scatter sorting (S)

All cells on the differentiation plate were dissociated to single cells by TrypLE treatment for 30 min, pelleted and resuspended in FACS buffer (7% FBS in 1X PBS) passed through a 70 μm cell strainer to get single cells and sorted using BD FACS Aria II cell sorter into scatter high and low fractions^18^.

### RPE yield calculation

When calculating RPE yield, the percentage of RPE in the starting culture was taken into account. The initial RPE (i-RPE) cell number before purification was estimated from the percentage PMEL17-positive population (PMEL17%) and the number of total cells in the mixed differentiation culture (N) using the formula (N x PMEL17%) / 100. The number of purified RPE (p-RPE) was determined by counting in a hemocytometer. All cells obtained from different purifications were considered as RPE, although the possibility of a minute fraction of non-RPE cells cannot be excluded. The percentage yield of RPE (RPE%) after purification would be (p-RPE/i-RPEi) × 100.

### PMEL17 flow cytometry

RPE cells purified from HDFa differentiation cultures and grown for 6 weeks, dissociated using TrypLE, fixed using IntraPrep Permeabilization Reagent (Beckman Coulter A07803) and stained overnight with PMEL17 antibody (1:1000, Melanosome Concentrate, M0634, Dako) with gentle rotation. the cells were stained with Alexa Fluor secondary antibody (1:1000, A31571, Thermo Fisher Scientific) and analyzed using BD LSR II Flow Cytometer.

### Immunocytochemistry

Purified RPE cells were grown on Transwells for 6 weeks fixed with 4% paraformaldehyde (pH 7.4) for 20 min at room temperature (RT), permeabilized with 0.2% Triton X-100 for 5 min and blocked with 1% bovine serum albumin (BSA, Sigma-Aldrich) in PBS for 1 h. Cells were then incubated with primary antibodies diluted in 1% BSA overnight at 4 °C. After washes with PBS, they were incubated with Alexa Fluor secondary antibodies (1:1000, A21202 and A10042, Thermo Fisher Scientific), DAPI, for 30 min at RT. Cells were mounted using Fluorsave (Merck Millipore 345789) and imaged using an LSM 700 confocal microscope (Zeiss). The primary antibody dilutions used in the study were ZO-1 (1:100, 617300, Thermo Fisher Scientific), PMEL17 (1:1000, M0634, Dako M0634), RPE65 (1:125, ab13826), Ezrin (1:500, ab4069).

### Transepithelial electrical resistance (TEER)

Cells were cultured on 24-well Transwells. TEER measurements were taken weekly, using EVOM2 Epithelial Volt Ohm meter (World Precision Instruments). Net TEER (Ω cm^2^) was calculated by subtracting the resistance values of experimental Transwells from the controls containing no cells and multiplying net values by the area of the Transwells (0.33 cm^2^).

### Photoreceptor outer segment (POS) phagocytosis assay

POS were isolated from porcine eyes as described previously^33,34^. RPE cells grown on Transwells for 6 weeks were challenged with FITC-labelled POS for 2 h at 37 °C, washed thrice with PBS and dissociated to single cells using TrypLE for 30 min. Cells challenged with unlabeled POS were used as controls. FITC fluorescence was measured using BD LSR II Flow Cytometer^28^.

### Statistical analysis

All experiments were performed in triplicate and statistical analyses were performed using GraphPad Prism. Comparisons between experiments were performed using the unpaired two-tailed students and statistical significance was established as an t-test (*) = p < 0.05; (**) = p < 0.005; (***) = p < 0.0005; (****) = p < 0.0001.

## Supplementary Information

Supplementary Information.

## Data Availability

The datasets used and/or analyzed during the current study are available on reasonable request.
