# Supplementary material for: Customized strategies for high-yield purification of retinal pigment epithelial cells differentiated from different stem cell sources
Source: Sci Rep. 2022 Sep 16;12:15563. doi: 10.1038/s41598-022-19777-2 (PMC9481580; doi:10.1038/s41598-022-19777-2)
Supplement: Supplementary file 1 — Supplementary Information. [file 41598_2022_19777_MOESM1_ESM.pdf]

Customized strategies for high-yield purification of retinal pigment epithelial cells differentiated from different stem cell sources

Kakkad Regha[1,2], Mayuri Bhargava[1,3], Abdurrahmaan Al-Mubaarak[1,2], Chou Chai [4], Bhav Harshad Parikh[1], Zengping Liu[1,2,5], Claudine See Wei Wong[1], Walter Hunziker[1,6], Kah Leong Lim[4], *Xinyi Su[1,2,3,5]

Supplementary Figure 1

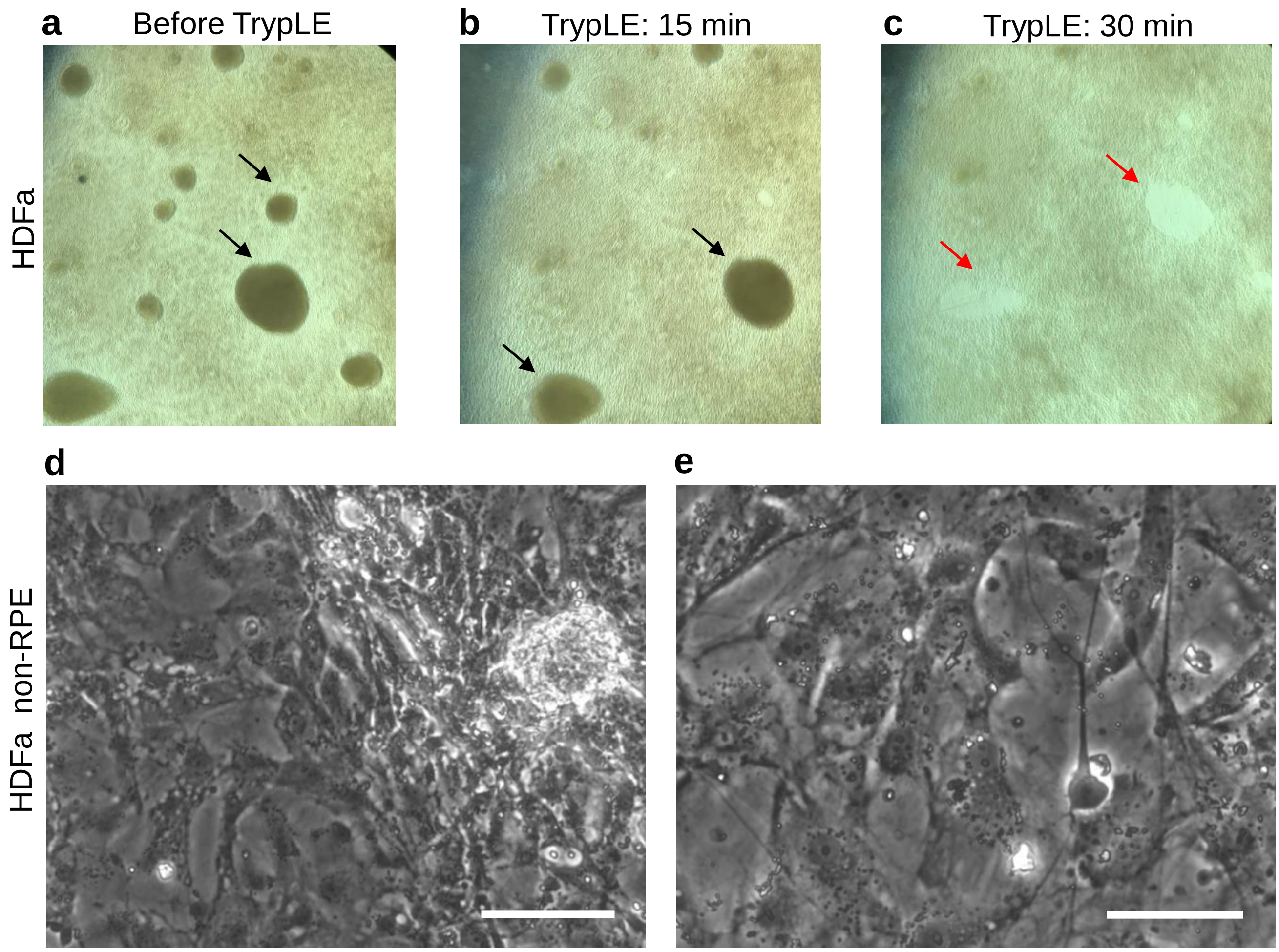

Supplementary Fig. 1: **RPE and non-RPE cells in HDFa differentiation cultures.** (**a** – **b**) 40X magnified images of dome-shaped non-RPE clusters (black arrows) scattered in RPE cell sheet at different stages of TrypLE purification. (**c**) Empty areas formed by the removal of non-RPE clusters (red arrows). (**d** – **e**) Non-RPE cells in HDFa differentiation cultures showing flat cells and clusters. Scale bar for (d) and (e), 100 μm.

Supplementary Figure 2

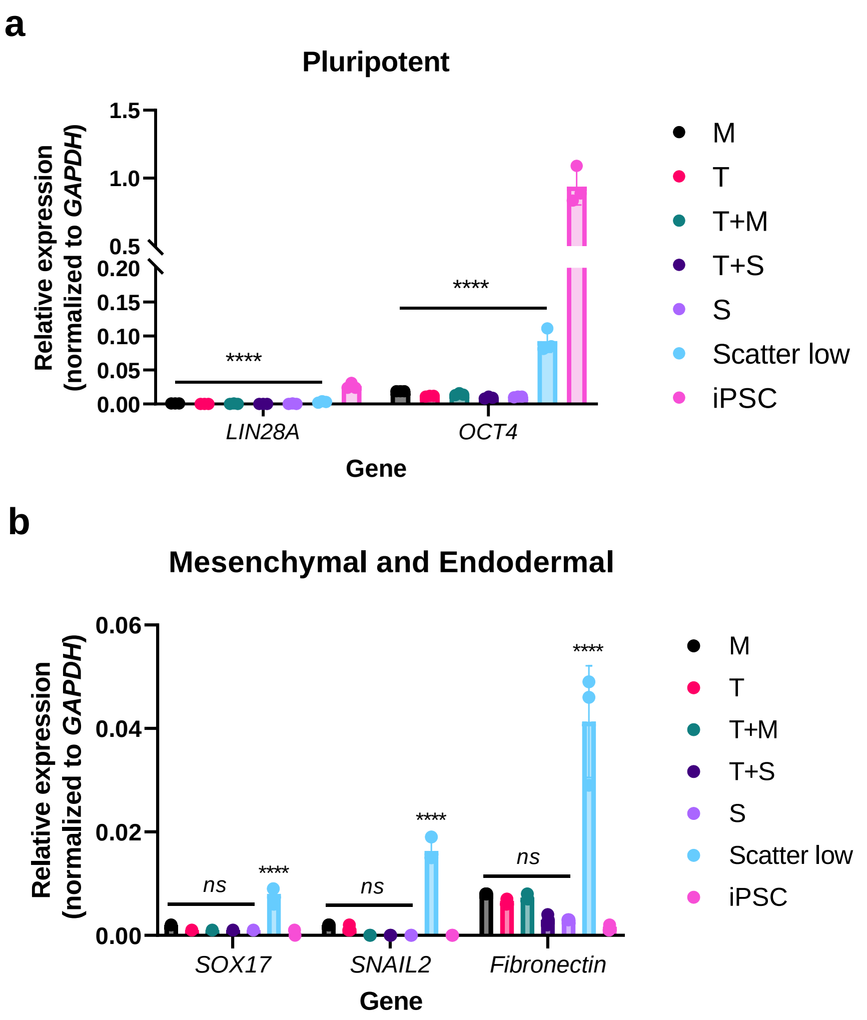

Supplementary Fig. 2: **RT-qPCR analysis of 6 weeks old HDFa-RPE cultures purified by different purification methods.** Markers specific for (**a**) pluripotent and (**b**) mesenchymal and endodermal cells. iPSC: HDFa stem cells. Scatter low: Cells obtained from scatter low fraction after scatter sorting and cultured for 6 weeks. Both (a) and (b) are compared against iPSC. Data represents mean ± s.d. of three replicates for RT-qPCR. Statistical analysis was done using one-way ANOVA, followed by Tukey's honest significance difference (HSD) *post hoc* test. (****)=$p<0.0001$.

Supplementary Table 1 | Primers used in this study

Gene	Sequence (5'→3')
*GAPDH*_Forward	CAGCCTCAAGATCATCAGCA
*GAPDH*_Reverse	TGTGGTCATGAGTCCTTCCA
*PMEL17*_Forward	GTTGATGGCTGTGGTCCTTG
*PMEL17*_Reverse	CAGTGACTGCTGCTATGTGG
*RPE65*_Forward	CCTGATTCATACCCATCAGAACCC
*RPE65*_Reverse	CACCACACTCAGAACTACACCATC
*OCT4*_Forward	CAGTGCCCGAAACCCACAC
*OCT4*_Reverse	GGAGACCCAGCAGCCTCAAA
*LIN28A*_Forward	GCGGGCATCTGTAAGTGGTT
*LIN28A*_Reverse	TGTGCAGCTTACTCTGGTGC
*Fibronectin*_Forward	GATGCAGACACAGAGCCAAA
*Fibronectin*_Reverse	CAGTCCTCAGTGGCAGATCA
*SNAIL2*_Forward	AGCATTTCAACGCCTCCA
*SNAIL2*_Reverse	GGATCTCTGGTTGTGGTATGAC
*SOX17*_Forward	ACGCTTTCATGGTGTGGGCTAAG
*SOX17*_Reverse	GTCAGCGCCTTCCACGACTTG
